# Impact of entrepreneurial orientation and risk sharing on organizational performance influencing role of news media and public opinion

**DOI:** 10.3389/fpsyg.2023.1126743

**Published:** 2023-02-09

**Authors:** Zhixiu Zhang, Yunwen Xing

**Affiliations:** ^1^School of Marxism, Shanxi University, Taiyuan, China; ^2^School of Marxism, Shanghai Jiao Tong University, Shanghai, China

**Keywords:** entrepreneurial orientation, creativity, innovativeness, proactiveness, autonomy, risk sharing, public opinion, organizational performance

## Abstract

An entrepreneurial orientation is a method of implementing a strategy that makes use of a variety of resources that are owned by organizations. Entrepreneurial orientation is one of the primary factors that led to the company's founding. Risk sharing is a useful tactic that can be implemented by businesses in order to mitigate the amount of risk to which they are exposed. As a consequence of this, the objective of the research is to ascertain how the performance of an enterprise can be affected by the presence of both an entrepreneurial orientation and shared risk. The proliferation of news media has led to modifications in the ways in which businesses carry out their day-to-day operations, which in turn has an effect on the overall success of the organization. As a direct consequence of this, the research looked into the function that the news media play as a moderator of the connections that exist between entrepreneurial orientation, risk sharing, and the level of performance achieved by organizations. Even for enormous, well-known businesses that are active on a global scale, damaging publicity has the potential to lower the value of their companies. The aim of this study was to investigate the impact of entrepreneurial orientation and risk sharing on organizational performance with the mediating role of news media and moderating role of public opinion. In order to achieve the objective of the study, a quantitative research approach was utilized. Data were collected from 450 managers of SMEs with the help of a questionnaire that was adapted from previous studies. A simple random sampling technique was used to collect data. The findings of the study showed that the relationship between entrepreneurial orientation, risk sharing, and organizational performance is positive and significant. The findings also showed that news media significantly mediated this relationship and public opinion moderated the relationship between news media and organizational performance. The current study has some practical and managerial implications which help SMEs to increase their performance.

## 1. Introduction

Entrepreneurship has become a key part of economies, and having an entrepreneurial orientation is essential for success. The authors defined entrepreneurial orientation as the process, practices, and decision-making activities that leads to a new entry (Kreiser et al., 2002; Rauch et al., 2009; Wales et al., 2013). Entrepreneurial decision-making practices, methods, and styles are portrayed due to firm's strategic orientation. Another researcher similarly described that the above are closely related to the strategic decision-making process and strategic management (Cui, 2021). Decision-making activities, practices, and strategic orientations reflect directions of behaviors chosen by entrepreneurs (Bouncken et al., 2020). Research on entrepreneurial orientation has remarkably grown in the international business research arena. Entrepreneurial orientation has also been the basis for several studies in many countries (Nor-Aishah et al., 2020). Although many business owners were frustrated as they were motivated to use their knowledge and experience in running businesses to turn their losses into positive returns (Yin et al., 2020). It is a widely held belief that small and medium-sized firms, sometimes referred to as SMEs, make a substantial contribution to the expansion of a nation's economy (Lumpkin and Dess, 1996; Ripollés-Meliá et al., 2007; Kropp et al., 2008). Multiple governments, particularly in underdeveloped or developing countries, make an effort to support and boost the development of the small and medium enterprise (SME) sector to achieve developmental goals and address the challenge of unemployment. This is done to achieve economic growth and address the problem of joblessness (Man and Lau, 2005).

The number of small and medium-sized enterprises (SMEs) in China accounts for more than 90 percent of the total number of businesses in the country. Additionally, SMEs are responsible for more than 50 percent of the country's tax revenue, more than 60 percent of GDP, and more than 80 percent of urban employment (Basso et al., 2009; Bolton and Lane, 2012; Wales, 2016). The growth of small and medium-sized enterprises (SMEs) is extremely important to policymakers because of their large size and their ability to generate synergies. As of right now, the economy of China has entered the stage of high-quality development; consequently, encouraging the high-quality development of SMEs can assist the economy in becoming more successful in its pursuit of high-quality development (Lee and Peterson, 2000; Keh et al., 2007; Moreno and Casillas, 2008; Gupta and Gupta, 2015). They are primarily responsible for the employment that is generated in the nation. The small and medium-sized business sector is often referred to as the “engine of economic growth.” According to the available research, there is no single definition of a small and medium-sized enterprise (SME) that applies not only in China but also elsewhere in the world. Multiple governmental institutions and governments all over the world each have their own definition of what constitutes a small or medium-sized business (Ali Khan et al., 2021).

Small businesses and projects typically revolve around a single leader or executive, in contrast to medium- and large-scale ventures, which create a more structured managerial team to manage or run a project (Wiklund, 1999; Lumpkin et al., 2009; Todorovic et al., 2011). For this reason, the research will primarily focus on small businesses (Rezaei and Ortt, 2018). The rationale for this research's decision to prioritize small businesses as its primary subject matter is as follows: According to Ali Khan et al. (2021) Within 6 years of their launch, 60% of entrepreneurial enterprises fail, and one of the main causes of this failure is the lack of financial resources or managerial skills on the side of the leaders or entrepreneurs. According to Rezaei and Ortt (2018), For a variety of factors, such as a lack of business-friendly policies, improved infrastructure, good economic conditions, and greater entrepreneurial orientation, as well as corruption and high operating costs, 40% of new enterprises fail before their third anniversary (Galbreath et al., 2020). Typically, entrepreneurial approach is judged to have a favorable impact on an organization's success. Regarding the research gap, studies have been done using entrepreneurial orientation as a latent construct in opposition to the goal of figuring out the performance level of SMEs. Despite this, relatively little research has been done on the aspects of entrepreneurial orientation to assess the performance of small firms, especially during the challenging years of COVID-19 (Miles and Arnold, 1991; Covin and Lumpkin, 2011; Covin and Miller, 2014). The results of this investigation will therefore be completely unique in this context. The role that small firms play in an economy's growth is well-known and well-acknowledged. Even the majority of already developed economies seem to rely greatly on the success of SMEs for their own economic development (Al-Mamary and Alshallaqi, 2022). It is vital to note that the lack of skill sets needed to conduct entrepreneurial initiatives makes it difficult for small enterprises to endure for extended periods of time. This makes it difficult for small firms to remain in operation. Because of this, they are both equally interested in finding out what causes small firms to fail. Regarding the practical ramifications, the research of entrepreneurial orientation in connection to performance is of the utmost importance. The performance of a company is significantly influenced by entrepreneurial orientation; the current study examines both of these crucial elements with respect to China.

One of the difficulties that entrepreneurs of small businesses face in developing nations is the difficulty of gaining timely access to information that is crucial to the success of their companies (Falahat et al., 2022). In China, a significant amount of effort is put into encouraging and supporting the development of startup entrepreneurs in small businesses. Building relationships and gathering information are the two aspects of development that have the most significant impact on the various business-related facets of the process (Dogan et al., 2022). Small businesses have historically relied heavily on personal connections with members of both the business community and the government in order to obtain pertinent information (Hernández-Perlines, 2016). Activities related to risk management may consist of avoiding, reducing, transferring, sharing, or even taking the risk (Cottle Hunt and Caliendo, 2022). This is done with the goal of lowering the likelihood of an incident occurring as well as its potential repercussions (Denuit et al., 2022). Risks in a relationship involving a supply chain can be reduced by entering into pre-crisis agreements and working together more effectively (Hajko et al., 2018). A poor image in the eyes of the public can be detrimental to a company's bottom line and overall performance. The public is more likely to pay attention to negative information in comparison to positive information, and they are also more likely to base their decisions on negative information (Gorodnichenko et al., 2021).

It is essential to address the challenges that are associated with the achievement of success by SMEs. The model of entrepreneurial orientation, risk sharing, news media, and public opinion is put through its empirical paces in this study (Dogan et al., 2021). Entrepreneurial orientation is measured through four dimensions. This study will also be helpful for start-ups in understanding and learning relevant vital tools and skills that could help improve and drive their performance. These tools and skills can be found in this study (Ali Khan et al., 2021). In addition to this, the study sheds light on the application of covariance-based structural equation modeling to the process of developing and validating the model that was derived from the aims of the research.

Individual and organizational success significantly impacted by one's propensity to pursue entrepreneurial opportunities (Gede et al., 2020). According to Rezaei and Ortt (2018), a high entrepreneurial orientation is related with higher performance in new ventures, whereas a low entrepreneurial orientation is associated with bad financial performance. As a result, it is essential for managers as well as investors to have a deeper comprehension of the connection that exists between an entrepreneurial approach and the performance of an organization. Previous research has made attempts to investigate the relationships between entrepreneurial orientation and firm performance utilizing a variety of measures of organizational performance including market valuation, total return, and accounting profitability as some of the indicators of firm performance. Nevertheless, these studies were carried out mostly with employee-owned enterprises or in the setting of economies that were still in the process of emerging.

The purpose of this study, which takes into account the significance of factors such as entrepreneurial orientation, risk sharing, news media, public opinion, and firm performance, is to: (i) Determine the nature of the connection between entrepreneurial orientation and risk sharing and the overall performance of small businesses. (ii) Investigate the impact that the news media has on the performance of small businesses. (iii) Investigate the possibility that the news media play a role in mediating the relationship between entrepreneurial orientation, risk sharing, and the performance of small businesses. (iv) Investigate the possibility that public opinion plays a moderating role in the connection between the news media and the performance of small businesses. The study has been distributed as the first sections defines the introduction, In the second section, we begin by conducting a literature review on the effect of entrepreneurial orientation and risk sharing on firm performance. We then demonstrate that there are a variety of variables that can both mediate and moderate the relationship between entrepreneurial orientation and firm performance, as well as the relationship between risk sharing and firm performance.

## 2. Literature review

Research on business and economic growth frequently focuses on entrepreneurship as one of its primary areas of study. As a result of the fact that this study investigates the connection that exists between business performance and an entrepreneurial orientation, as well as the role that entrepreneurial competencies play in mediating this connection, it has been found that the “Resource-Based View” is the theoretical framework that most closely corresponds to the findings of this study (RBV). RBV states that a resource, whether it be tangible or intangible, should be “valuable, rare, inimitable, and organization.” According to Barney et al. (2001), RBV views intangible assets as characteristics of human capital, more specifically skills. Entrepreneurial orientation is a notions that is unique to each individual, making it difficult for competitors to imitate them. In this particular setting, RBV can be implemented, and so can entrepreneurial orientations and competencies. These are the kinds of skill sets that can bring an organization to a greater level of performance (Ali Khan et al., 2021).

### 2.1. Entrepreneurial orientation

In previous studies, the idea of coordination toward entrepreneurial association has been assigned a plethora of labels, including entrepreneurial orientation, proclivity, smartness, and intensity, as well as corporate entrepreneurship. These labels have been used to categories the degree to which an individual engages in entrepreneurial activity (Galbreath et al., 2020), “a corporation's strategic approach toward entrepreneurship,” or “entrepreneurial Orientation,” is what the acronym. Theoretically, the term “Entrepreneurial Organization” (EO) refers to fundamental tactics and procedures for the improvement of entrepreneurial activities and decisions, in addition to the ways that decision makers employ to raise the tenacity of their firms, upkeep, and overall operations (Abubakar et al., 2019).

EO to be a variable that was made up of three distinct dimensions: innovativeness, risk-taking, and proactiveness (Ashwyn Bapoo et al., 2021). For an EO to be manifested, all three of these dimensions needed to positively co-vary with one another. Nevertheless, included two additional characteristics in EO. These two new dimensions are called autonomy and aggressiveness. In order to be innovative, businesses need to generate new ideas, improve existing ones, and keep an eye out for new business prospects (Mathafena and Msimango-Galawe, 2022). It comprises a propensity to participate in innovativeness and research through creative work, Research and Development (R&D), and other forms of research and development (Nawaz and Guribie, 2022; Sandra Marcelline et al., 2022). The aspect of taking risks is extremely close to that of creativity and originality. This includes daring behaviors such as delving into the unknown or committing considerable resources to the possibility of outcomes that are uncertain. This dimension includes business risks, common hazards, fundamental leadership risks, decision making risks, and decision making risks related to business (Baki et al., 2018).

### 2.2. Entrepreneurial orientation and organizational performance

According to Gede et al. (2020), the performance implications of entrepreneurial orientation are the largest stream of research within the field of entrepreneurship. The predominant argument is that entrepreneurial orientation plays an important role in enhancing a firm's performance (Lurtz and Kreutzer, 2016). Anderson et al. (2015) found that entrepreneurial orientation motivates companies to aggressively launch product innovations, explore opportunities and favor new product development activities. Rezaei and Ortt (2018) noted that entrepreneurial orientation contributes to the success of new products by allowing firms to identify and proactively take advantage of new business opportunities.

When we talk about creativity, what we're referring to is the process through which individuals come up with ideas that are unique and have the potential to be beneficial to the company (Hughes and Morgan, 2007). It is possible for persons working in any job to develop it, and it can range from suggestions for minor adjustments to ideas for significant advances in the field (Haider and Tehseen, 2022). On the other hand, innovation is defined as “any thought, activity, or tangible item considered to be novel by the relevant unit of adoption” (Ullah and Danish, 2020). According to Ordieres-Meré et al. (2020), innovativeness has a powerful and positively connected relationship with capabilities that are founded on competencies. It has been observed that businesspeople who possess specific skill sets based on competencies are regarded as inventive and have the potential to function more effectively for their small companies (Cui et al., 2018). This is especially true for businesspeople who have been in the industry for a longer period. Creativity and innovativeness are said to have a substantial association with an individual's potential to be an entrepreneur, according to one school of thought (Balsalobre-Lorente et al., 2019). Based on the above discussion following hypothesis has been developed.

*H1: Creativity has a significant and positive impact on organizational performance*.
*H2: Innovativeness has a significant and positive impact on organizational performance*.
*H3: Pro-activeness has a significant and positive impact on organizational performance*.
*H4: Autonomy has a significant and positive impact on organizational performance*.

### 2.3. Risk sharing and organizational performance

The term “risk sharing” refers to the tendency to choose unique methods of carrying out tasks or to depart from well-established patterns in order to pursue unforeseeable results (Denuit et al., 2022). In another piece of research that Asbari et al. (2020) carried out, the researchers came to the conclusion that an individual's tendency to share risks is one of the most reliable indicators of entrepreneurial behavior and has a direct correlation with competencies. According to the findings of a study that was carried out in China on 157 young business owners by Denuit et al. (2022), there is a positive relationship between a firm's performance and a risk-sharing attitude. On the other hand, Wang and Neihart (2015) evaluated the association between risk sharing and firm performance using data from 60 stock exchange enterprises. The findings of the study indicated that there was a correlation between the two variables, but it was not very significant. In a different piece of research, Glaser et al. (2016) carried out an investigation on 383 middle-level managers who were employed in a total of 34 distinct company departments. Their findings also demonstrated a correlation between the firm's performance and risk-sharing behavior, Furthermore, according to Tarafdar et al. (2010), the presence of entrepreneurs who are willing to share risks is associated with improved SME performance in developing economies. Peng et al. (2020) conducted a study that was very similar to this one on 381 SMEs in Nigeria using the SEM model in order to determine the relationship between risk-sharing propensity and SMEs performance.

*H5: Risk sharing has a significant and positive impact on organizational performance*.

### 2.4. News media

Even though there is constant debate regarding the positive and negative consequences of news media, the majority of businesses are still unsure whether or not they will utilize news media. In light of this, we conducted study to investigate the connections between the use of news media and the performance of organizations. Integrating and making use of news media has a significant and beneficial impact on the performance of businesses in terms of accelerating change, cutting costs, and developing new products and services (Apuke and Omar, 2021). In a similar vein, adoption of news media platforms by businesses comes with a number of advantages, and some researchers have discovered a substantial connection between organizational media usage and the success of businesses (Masri and Jaaron, 2017). It has been suggested that the media should be considered a relevant corporate governance mechanism because of its essential role as a channel through which information is distributed to investors. Therefore, the media may serve as an information intermediary between companies and outside investors, thereby increasing the level of transparency and enhancing the level of interest protection afforded to outside parties (Bakir and McStay, 2018). The media select, analyze, and communicate earnings data, and they have the ability to influence how informative the data actually is. They have the potential to play a pivotal role in controlling the actions of dominant owners and directors through the reduction of informational asymmetries between internal and external agents and through the manipulation of the image and reputation of internal agents (Domenico et al., 2021). The media has an incentive to maintain a good reputation by providing information that is accurate and credible, especially in a context in which competition is intense. According to Shehata and Strömbäck (2021), the work done by the media lowers the costs of dealing with the rational ignorance paradox. This paradox occurs when the costs of being well-informed exceed the benefits obtained from the information. The costs of dealing with the rational ignorance paradox are reduced because of the work done by the media. Disclosure of information through the media enables outside investors to avoid the risks associated with rational ignorance.

*H6*: *News media has a significant and positive impact on organizational performance*.
*H7: News Media mediated the relationship between creativity and organizational performance*.
*H8: News Media mediated the relationship between innovativeness and organizational performance*.
*H9: News Media mediated the relationship between pro-activeness and organizational performance*.
*H10: News Media mediated the relationship between autonomy and organizational performance*.
*H11: News Media mediated the relationship between risk sharing and organizational performance*.

### 2.5. Public opinion

Public opinion is looking at how the social environment influences organizational leaders as part of a larger study agenda, and one of the components of this agenda is public opinion. When there is no room for interpretation, public opinion has an effect on decisions on policy, the opinions of the Supreme Court (despite the fact that Justices are appointed for life), the decision to go to war, as well as the proposal and approval of measures (Hu and Li, 2018). “Public opinion” is defined as “the opinion held by the majority and the opinion that the minority acquiesces in passively.” It is impossible for there to be a public opinion when the opinion of the majority is directly opposed by the opinion of the minority. If the opinion is harmful to the interests of one group or comes at the expense of one community, then it cannot be considered the opinion of the public. The pursuit of the public good should be the objective of public opinion. Even though it may be the viewpoint of the most enlightened segment of the population, it still needs to be capable of promoting the general welfare of everyone (Gorodnichenko et al., 2021). The fundamental idea that has been put forward to explain this phenomenon is that in a democratic society, elected officials attempt to earn re-election by demonstrating that they are sensitive to the needs of the people they represent. Now, there is a growing body of research coming from areas outside of politics that demonstrates the desire to win the approval of the general public is a more important component than the desire to be re-elected (Ahmed et al., 2015).

*H12: Public opinion moderated the relationship between news media and organizational performance*.

### 2.6. Conceptual framework

**Figure 1:**
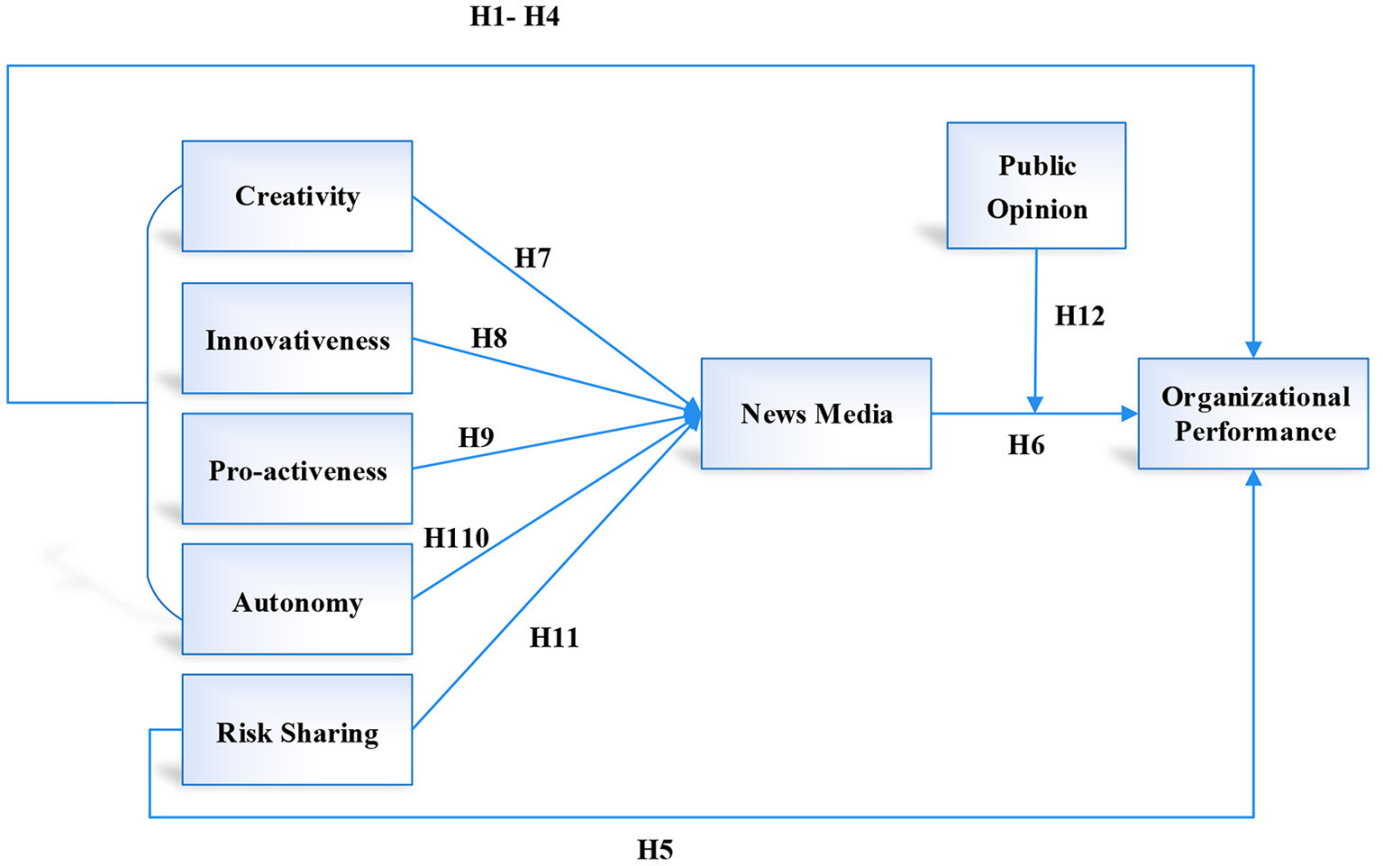
Conceptual framework.

According to the research that has been conducted, entrepreneurial orientation is comprised of four different dimensions, namely autonomy, proactivity, creativity, and innovativeness. On the other hand, risk sharing and organizational performance have been operationalized through the use of perceptual measures. The news media is being tested as a mediator variable, while public opinion is being tested as a moderator variable. In this study, a model that claims a connection between entrepreneurial orientation, risk sharing, news media, public opinion, and organizational performance is tested. In addition, the role of news media as a potential mediator of the relationship between entrepreneurial orientation, risk sharing, and organizational performance has been tested in the context of small and medium-sized enterprises (SMEs) in China. Finally, the moderating role of public opinion between the relationship of entrepreneurial orientation, risk sharing, and organizational performance has also been tested. There are eight hypotheses pertaining to entrepreneurial orientation dimensions have been developed to test relationships with news media and organizational performance as discussed in the literature; two hypotheses have been developed to test the relationship of risk sharing news media and firm performance; five hypotheses have been developed to test the mediating effects of news media with each dimension of entrepreneurial orientation, risk sharing, and organizational performance; and eight hypotheses have been developed to test whether or not there is a relationship between entrepreneurial orientation and risk sharing. This is represented in Figure 1, which is the conceptual framework.

## 3. Methodology

### 3.1. Study design

Data were collected from managers of small and medium-sized enterprises (SMEs) located in China so that the study could achieve its objectives. For this reason, a questionnaire was used that the respondents could self-administer when they had some free time available to them. According to the findings of Sarstedt et al. (2014), an adequate sample size for statistical analysis would consist of at least 10–20 times more variables than were employed in the research. In a similar vein (Hair et al., 2006; Leguina, 2015), suggested that the minimum sample size might be determined by multiplying 10 by the total number of arrowheads that were pointing in the direction of the endogenous construct. As a result, the minimum number of participants needed for the current investigation is 180, which seems to be adequate for statistical analysis given that there are nine arrowheads pointing in the direction of the endogenous construct in this study. The author posted all of the questions on the author's Wechat account and shared them with the various groups. Before any of the participants were asked to fill out the questionnaire, they were all provided with information regarding the objectives of the research and assurances regarding the confidentiality of their responses and participation in the study. Data were collected from September 2022 to November 2022. The participants received the questionnaire at random during the course of the study. In the end, a total of 400 samples were collected, and after discarding any questionnaires that were either incomplete or contradictory, data analysis was performed on the 367 samples. A demographic profile of the respondents is provided in Table 1, which is given below. This profile includes the gender, age, education level, experience, and whether or not the participants listen to the news.

**Table 1:** Demographic profile of the respondents.

**Demographic item**		**Frequency**
Gender	Male	200
	Female	167
Age	20–30 years	59
	31–40 years	53
	41–50 years	105
	More than 50 years	150
Education	Bachelor	186
	Master	111
	M.phil./Ph.D.	70
Experience	1–3 Years	98
	3–5 Years	245
	More than 5 years	24
Time spend of social media	Daily	290
	Weekly	52
	Once in a month	25

### 3.2. Measure

The questionnaires for each variable including entrepreneurial orientation, risk sharing, organizational performance, news media, and public opinion were adapted from those used in previously conducted research. In order to evaluate the entrepreneurial orientation, dimensions such as creativity (six items), innovativeness (five items), proactiveness (four items), and autonomy (five items) were adopted from Ali Khan et al. (2021). The scale of organizational performance was adopted from Galbreath et al. (2020), the scale of news media was adopted from Peña-Martel et al. (2018), and the scale of public opinion was adopted from Gorodnichenko et al. (2021). The items that measure risk sharing were adapted from the scale that was proposed by Cottle Hunt and Caliendo (2022). In addition, this investigation made use of the Likert scale, which consists of five levels and ranges from 1 (which denotes “not at all”) to 5 (which denotes “very much”) to evaluate each component of entrepreneurial orientation as well as risk sharing, news media, public opinion and organizational performance. In the run-up to the distribution of the questionnaire, we had enlisted the help of two psychologists so that we could analyze and adjust the way in which the scale was presented. The questionnaire underwent a pilot test with a select group of 20 individuals (managers of SMEs), after which it was refined based on the responses that were received based on the results of the pilot test. The questionnaires that were filled out during the pilot project were not utilized in the analysis that was carried out in the end.

### 3.3. Common method bias

The bias that is produced by CMV is known as the common method bias, and it manifests itself when the estimated relationship between one construct and another might be exaggerated; to put it another way, CMV generates a systematic covariation that is in excess of the true relationship that exists between the scale items. As a consequence of this, the altered values of the observed correlations and of other relevant indicators might lead to either incorrect estimates of the reliability and convergent validity constructs in the study or erroneous parameter estimates related to the magnitude and significance of the relationships among constructs. Both of these outcomes would be a negative consequence of the altered values of the observed correlations and of other relevant indicators (Rodríguez-Ardura et al., 2020). If the total variance retrieved by one element is >50% of the overall variance, then your research suffers from common method bias. Since the total variance recovered by one component is 25.440%, which is less than the suggested threshold of 50%, there is no concern with common method bias in these data. Table 2 shows the result of CMV.

**Table 2:** Common method bias.

**Factor**	**Initial eigenvalues**			**Extraction sums of squared loadings**		
	**Total**	**% of variance**	**Cumulative %**	**Total**	**% of variance**	**Cumulative %**
**Total variance explained**						
1	10.873	27.182	27.182	10.176	25.440	25.440
2	7.119	17.798	44.980			
3	4.381	10.953	55.933			
4	1.989	4.972	60.905			
5	1.356	3.389	64.294			
6	1.251	3.128	67.422			
7	1.205	3.012	70.434			
8	0.882	2.206	72.639			
9	0.813	2.033	74.672			
10	0.698	1.746	76.418			
11	0.668	1.671	78.088			
12	0.625	1.563	79.652			
13	0.550	1.374	81.026			
14	0.539	1.347	82.373			
15	0.522	1.304	83.676			
16	0.458	1.145	84.821			
17	0.447	1.118	85.939			
18	0.426	1.064	87.003			
19	0.383	0.958	87.961			
20	0.358	0.895	88.856			
21	0.337	0.844	89.699			
22	0.331	0.828	90.527			
23	0.311	0.776	91.303			
24	0.303	0.758	92.062			
25	0.294	0.735	92.797			
26	0.274	0.685	93.481			
27	0.262	0.654	94.136			
28	0.257	0.644	94.779			
29	0.241	0.602	95.382			
30	0.237	0.592	95.974			
31	0.216	0.539	96.513			
32	0.205	0.512	97.025			
33	0.199	0.497	97.521			
34	0.184	0.460	97.981			
35	0.177	0.444	98.424			
36	0.145	0.362	98.786			
37	0.142	0.356	99.142			
38	0.130	0.324	99.466			
39	0.115	0.287	99.753			
40	0.099	0.247	100.000			

Extraction Method: Principal Axis Factoring.

## 4. Data analysis and results

This study validated the model by using the partial least square structural equation modeling (PLS-SEM) method that is included in Smart-PLS 3.0. This method was used to analyze the data. PLS-SEM is better suited for exploratory studies than covariance-base structural equation modeling, which is one of the reasons it was chosen rather than covariance-base structural equation modeling. Another reason is that PLS-SEM is easier to interpret than covariance-base structural equation modeling. The initial point to make is that this study incorporates an exploratory analysis. Second, the PLS technique is suitable for the analysis of data derived from relatively small samples as a result of the flexibility it possesses.

### 4.1. Measurement model

**Figure 2:**
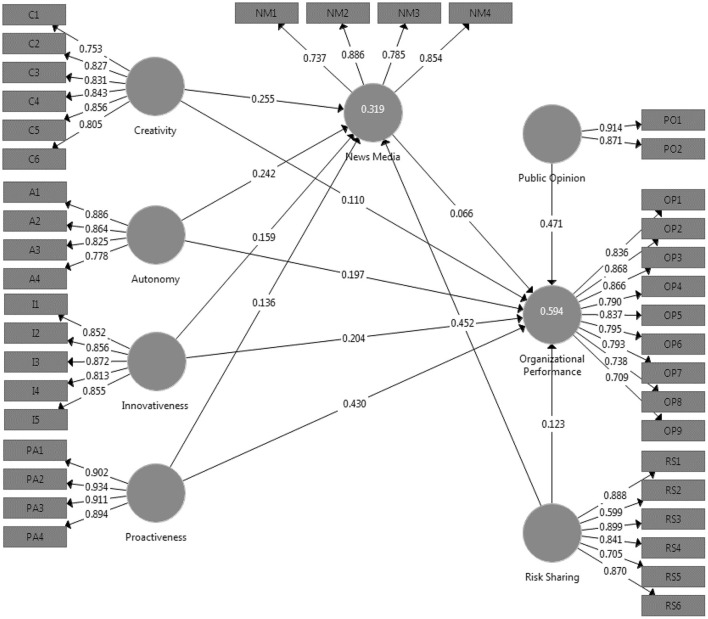
Structural model.

**Figure 3:**
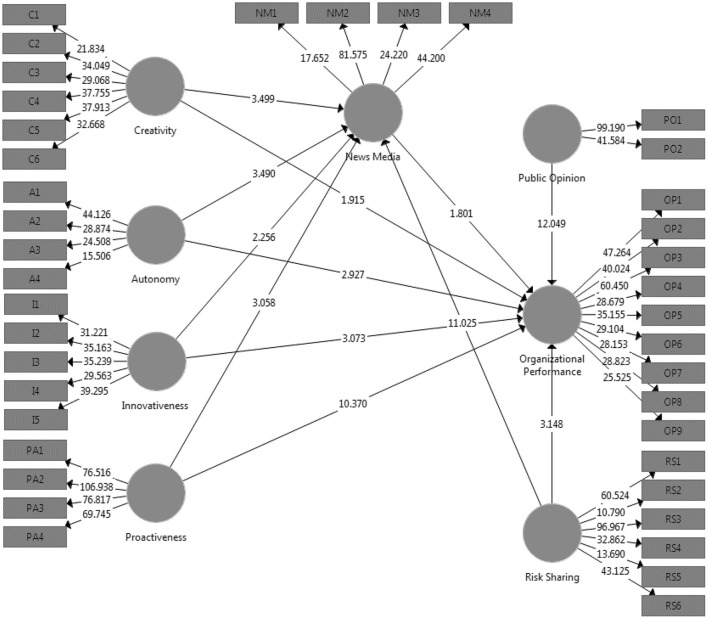
Measurement model.

In the context of measurement models, both the reliability of the model and its validity are important considerations to take into account. Cronbach's alpha, roh-A, composite reliability, and average variance extract were utilized in this investigation in order to assess the degree to which the model could be relied upon. In addition, convergent and discriminant validity were utilized in an analysis to determine the model's soundness (Hair et al., 2016). Table 3 and Figure 2 present the findings of the models used in this research to analyses the dependent relationships among all of the variables. To begin, the Cronbach alpha requires that it have a value that is >0.70 in order for it to be considered satisfactory (Avotra et al., 2021; Yingfei et al., 2021). Overall, the values of Cronbach's alpha for the model variables in this research are >0.70. For instance, the values of IVs (creativity, innovativeness, proactiveness, autonomy, and risk sharing), DV (organizational performance), mediator (news media), and moderators (public opinion,) are 0.861, 0.902, 0.904, 0.834, 0.932, 0.932, 0.748, and 0.890, respectively. These values are presented in Table 3. The Cronbach alpha threshold was provided, and these are the values that correspond to it. As a direct consequence of this, all values are recognized. Second, the roh-A values of all the variables have been modified in such a way that they now align with the threshold value. The third stage of the analysis involves looking into the composite reliability (CR) and average variance extract (AVE) of the model variables. Both the average variance extract and the acceptable values of the variables for composite reliability are >0.5, and the acceptable values of the variables are >0.7. The acceptable values for the variables are also higher than 0.5. In addition to this, the outer loadings of each variable were investigated, and the findings are presented in Table 2. When it comes to determining the appropriate outside loadings for various objects, a value that is >0.6 is considered appropriate (Figure 3). Every single item in the variables has a value that is >0.6.

**Table 3:** Construct reliability and validity.

	**Items**	**Outer loading**	**VIF**	**Cronbach's alpha**	**rho_A**	**CR**	**AVE**
Autonomy	A1	0.886	2.322	0.861	0.892	0.905	0.704
	A2	0.864	2.501				
	A3	0.825	1.837				
	A4	0.778	1.787				
Creativity	C1	0.753	1.739	0.902	0.902	0.925	0.672
	C2	0.827	2.308				
	C3	0.831	2.280				
	C4	0.843	2.619				
	C5	0.856	2.671				
	C6	0.805	2.208				
Innovativeness	I1	0.852	3.258	0.904	0.911	0.928	0.722
	I2	0.856	3.138				
	I3	0.872	2.817				
	I4	0.813	1.952				
	I5	0.855	2.333				
News media	NM1	0.737	1.639	0.834	0.861	0.889	0.668
	NM2	0.886	2.372				
	NM3	0.785	1.888				
	NM4	0.854	2.059				
Organizational performance	OP1	0.836	2.894	0.932	0.932	0.943	0.648
	OP2	0.868	3.642				
	OP3	0.866	3.665				
	OP4	0.790	2.489				
	OP5	0.837	2.925				
	OP6	0.795	2.612				
	OP7	0.793	2.500				
	OP8	0.738	2.525				
	OP9	0.709	2.332				
Proactiveness	PA1	0.902	3.164	0.931	0.932	0.951	0.829
	PA2	0.934	4.559				
	PA3	0.911	3.721				
	PA4	0.894	2.926				
Public opinion	PO1	0.914	1.556	0.748	0.767	0.887	0.797
	PO2	0.871	1.556				
Risk sharing	RS1	0.888	3.343	0.890	0.915	0.917	0.653
	RS2	0.599	1.414				
	RS3	0.899	4.310				
	RS4	0.841	2.484				
	RS5	0.705	1.608				
	RS6	0.870	3.199				

In addition, the collinearity issue was investigated by employing the variance inflation factor in the course of this study (VIF). Readings of the VIF that are lower than 0.5, as recommended by the researchers, are considered to be satisfactory (Hair et al., 2014). According to Table 3, the VIF values of the study model's core constructs range anywhere from 1.414 to 4.559. This range covers a wide variety of possible outcomes. It is evidence that the VIF values of all of the components meet the prerequisites set forth by the threshold. As a result, the research model that was utilized for this study did not exhibit any signs of having a problem with collinearity.

The Fornell-Larcker criterion and the heterotrait-monotrait (HTMT) method were utilized (see Table 4) in order to conduct an analysis of the discriminant validity of this research (Hair et al., 2016). The validity of the discriminant function can be checked using the Fornell-Larcker criterion by taking the square root of the average variance extract values for all model variables (Hair et al., 2016). Table 5 provides a comprehensive analysis of the discriminant validity of each variable, utilizing the Fornell-Larcker criterion as its primary reference point. Because the initial values of all variables within each column show the highest values relative to their subsequent values, this table demonstrates that the model's discriminant validity has been achieved (Hair et al., 2016).

**Table 4:** Discriminant validity (HTMT).

	**A**	**C**	**I**	**NM**	**OP**	**PA**	**PO**	**RS**
Autonomy							
Creativity	0.874							
Innovativeness	0.861	0.788						
News media	0.210	0.336	0.267					
Organizational performance	0.149	0.136	0.086	0.146				
Proactiveness	0.218	0.292	0.240	0.269	0.678			
Public opinion	0.215	0.293	0.267	0.208	0.767	0.622		
Risk sharing	0.200	0.190	0.114	0.539	0.175	0.110	0.082	

**Table 5:** Discriminant validity (Fornell-Larcker).

	**A**	**C**	**I**	**NM**	**OP**	**PA**	**PO**	**RS**
Autonomy	0.839							
Creativity	−0.769	0.820						
Innovativeness	−0.744	0.706	0.850					
News media	−0.183	0.295	0.236	0.817				
Organizational performance	0.136	−0.127	−0.060	−0.133	0.805			
Proactiveness	−0.198	0.269	0.223	0.240	−0.637	0.910		
Public opinion	0.170	−0.239	−0.219	−0.173	0.655	−0.523	0.893	
Risk sharing	−0.185	0.173	0.105	0.483	−0.165	0.107	−0.067	0.808

According to the HTMT rations criterion, in order for any of the variable values to be considered appropriate, they must have a value that is lower than 0.85. Despite this, HTMT scores of up to 0.90 are sometimes considered to be acceptable (Hair et al., 2016). The findings of this investigation are detailed in Table 5, where it is clear that each value lies within the permissible range, which extends from 0.85 to 0.90 and can accommodate all of the given options. The results of this investigation showed that the model that was proposed for the investigation has discriminant validity.

When the R2 score is >0.5, it is determined that the strength of the model in the initial data is strong. In this investigation, the level of model strength demonstrated by organizational performance (*R*^2^ = 0.594) was considered to be moderate (Hair et al., 2016). In addition to this, the values of *Q*^2^ for each of the latent constructs in the models are higher than Zero, which is a requirement for inclusion in the models. In addition to that, it functions as an example of significant signs. The values of *R*^2^ and *Q*^2^ are presented in Table 6.

**Table 6:** R-square values and Q-square values for the variables.

	***R* ^2^**	***Q* ^2^**
News media	0.319	0.193
Organizational performance	0.594	0.349

### 4.2. Direct path analysis

This study utilized a bootstrapping method with 5,000 different samples for the purpose of performing statistical validation on the model hypotheses (Hair et al., 2016). The *t* and *p*-values were analyzed in this study to determine whether or not the hypotheses should be accepted or rejected (Hair et al., 2016). The results of the H1 relationship, which predicted that creative thinking would have a significant impact on organizational performance, are broken down and explained in Table 7, which can be found here. Both the value of *t* and the value of *p* indicate that this proposal should be accepted (*t* = 1.9.15, *P* = 0.028). As a result, H1 is acceptable. The second hypothesis stated that the degree to which an organization is innovative has a significant bearing on the level of performance that it achieves. Both the value of t and the value of *p* indicate that this proposition should be accepted (*t* = 3.037, *P* = 0.001). Therefore, H2 can be accepted. The third hypothesis stated that the degree of proactiveness an organization possesses has a significant bearing on the performance of the organization. Both the value of t and the value of *p* indicate that this proposal should be accepted (*t* = 10.370, *p* = 0.000). As a result, H3 can be accepted. The fourth hypothesis stated that individual autonomy has a significant effect on the overall performance of an organization. The values of t and p point to the fact that this proposition will be accepted (*t* equals 2.927, and *P* equals 0.002). As a result, H4 is permitted. The fifth hypothesis stated that there is a significant impact that risk sharing has on the performance of an organization. Both the value of t and the value of p indicate that this proposition should be accepted (*t* = 3.148, *P* = 0.001). As a result, H5 is approved. In the sixth hypothesis, it was stated that the news media has a significant impact on the performance of organizations. Both the value of t and the value of p indicate that this proposal should be accepted (*t* = 1.810, *P* = 0.036). As a result, H6 is accepted.

**Table 7:** Direct effects.

**Hypotheses**	**Relationship**	**Beta**	**SD**	***T*-value**	***P*-values**	**Decision**
H1	Creativity -> organizational performance	0.110	0.058	1.915	**0.028**	Supported
H2	Innovativeness -> organizational performance	0.204	0.066	3.073	**0.001**	Supported
H3	Proactiveness -> organizational performance	0.430	0.041	10.370	**0.000**	Supported
H4	Autonomy -> organizational performance	0.197	0.067	2.927	**0.002**	Supported
H5	Risk sharing -> organizational performance	0.123	0.039	3.148	**0.001**	Supported
H6	News media -> organizational performance	0.066	0.037	1.801	**0.036**	Supported

Bold value shows the relationship and connection between the variables as significance.

### 4.3. Mediation analysis

In addition, the significance of the news media was investigated as a possible mediator in the relationship between creative output and organizational performance. The news media acts as a positive mediator of the relationship between creative output and organizational performance, as postulated in Hypothesis 7. The findings suggest that news media play a role as a mediator in the connection that exists between creative output and organizational performance (p = 0.042). As a consequence of this, the seventh hypothesis of this investigation is supported by the findings of the research. Table 7 presents the results of the investigation into the use of mediation. In a similar vein, Hypothesis 8 proposed that the news media acts as a positive mediator in the connection between innovativeness and organizational performance. The findings suggest that news media play a role as a mediator in the connection that exists between innovativeness and the performance of organizations (*p* = 0.023). As a consequence of this, the eighth hypothesis of this investigation is supported by the findings of the research. In addition, the ninth hypothesis of the research stated that news media acts as a positive mediator of the relationship between proactiveness and the performance of organizations. According to the findings, the role of a mediator in the connection between proactiveness and organizational performance (p=0.000) is played by the news media. As a consequence of this, the findings of the research lend credence to the ninth hypothesis that underlies this investigation. According to the study's tenth hypothesis, the relationship between autonomy and organizational performance is mediated by news media. This was one of the hypotheses that was tested. According to the results of the research, there is a significant correlation between autonomy and organizational performance (p = 0.008), and this correlation is positively mediated by the news media. As a result, H10 is also permitted. According to the study's eleventh hypothesis, the relationship between risk sharing and organizational performance is mediated by news media. This was one of the hypotheses that was tested. According to the results of the research, a connection exists between risk sharing and the performance of organizations, and that this connection is favorably mediated by the news media (p = 0.042). Consequently, H11 is also acceptable. Table 8 displays the findings of the mediation analysis that was performed.

**Table 8:** Indirect effects.

**Hypotheses**	**Structural paths**	**Path coefficient**	***T*-value**	***P*-value**	**Interpretation**	**Results**
H7	C -> NM -> OP	0.017	1.737	0.042	Partial mediation	Supported
H8	I -> NM -> OP	0.011	1.996	0.023	Partial mediation	Supported
H9	PA -> NM -> OP	0.009	8.841	0.000	Partial mediation	Supported
H10	A -> NM -> OP	0.016	2.425	0.008	Partial mediation	Supported
H11	RS -> NM -> OP	0.030	1.734	0.042	Partial mediation	Supported

### 4.4. Moderation analysis

**Figure 4:**
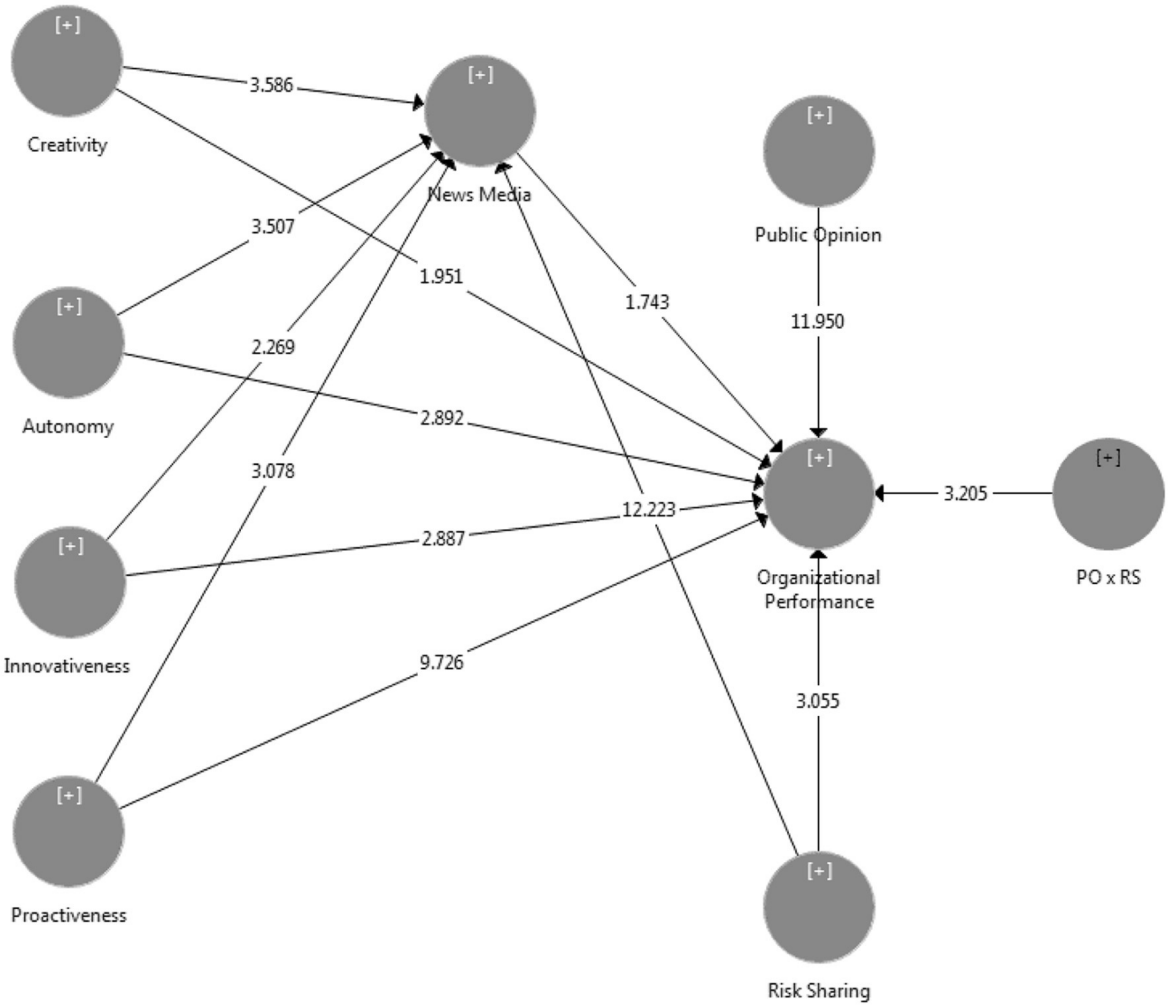
Public opinion as a moderator.

According to the twelfth hypothesis, the public's point of view acts as a significant moderating influence in the connection between the news media and the performance of organizations. An interaction term was utilized for the purpose of conducting an analysis that determined the degree to which public opinion served as a moderating factor. The findings of the research indicated that public opinion plays a moderating role in the connection that exists between the news media and the performance of organizations (*t* = 3.205, *p* = 0.000). As a result, solution H12 is approved. Figure 4 and Table 9 illustrate the moderating effect that public opinion has on the relationship.

**Table 9:** Moderation effect.

**Hypotheses**		**Original sample**	***T*-values**	***P*-values**
H12	AN x PE -> rumor sharing	0.030	3.205	0.000

## 5. Discussion

This study's objectives were to investigate the relationship between entrepreneurial orientation, risk sharing, and organizational performance; to determine whether or not news media fully or partially mediated this relationship; and to determine whether or not public opinion moderated the relationship between news media and organizational performance. In addition, the study sought to determine whether or not public opinion moderated the relationship between news media and organizational performance. The research was carried out on a group (*n* = 367) of Chinese SME managers currently employed in the country. This investigation, when it was all said and done, produced a number of important discoveries, which are as follows:

According to the hypotheses H1, H2, H3, and H4, the entrepreneurial orientation dimensions (also known as creativity, innovativeness, pro-activeness, and autonomy) have a significant effect on the performance of an organization. People who have a high entrepreneurial orientation are more likely to contribute toward performance, as shown by the findings of the study, which align with the findings of Ali Khan et al. (2021), who found that entrepreneurial orientation dimensions have a significant impact on organizational performance. The study found that creativity, innovativeness, pro-activeness, and autonomy have a significant and positive impact on organizational performance. This indicates that people with a high entrepreneurial orientation are more likely to contribute toward performance. The fifth hypothesis stated that the sharing of risks has a significant effect on the performance of an organization. The results of the research showed that sharing risks has a significant influence, both positively and significantly, on the performance of an organization. These findings are in line with the findings of a study that was conducted by Meekaewkunchorn et al. (2021), which stated that the performance of organizations increases when people share risk with the management. The news media has a significant impact on the performance of organizations, as stated in the fifth hypothesis (H5). The results of the study demonstrated that the news media has a constructive effect on the overall performance of the organization. These findings are in line with the findings of a study that was carried out by Verma et al. (2022), which stated that when people found positive news about an organization, it had a positive impact on stakeholders and employees, which caused performance to improve positively. These findings align with the findings of the study that was carried out.

In the hypotheses H7, H8, H9, H10, and H11, it was stated that the news media acted as a mediator between the relationship between organizational performance and creativity, innovativeness, proactiveness, autonomy, and risk sharing. According to the findings of the study, there is a significant amount of mediation taking place between the relationships between creativeness, innovativeness, pro-activeness, autonomy, risk sharing, and organizational performance and the news media. According to the findings of the mediation analysis, the hypotheses H7, H8, H9, H10, and H11 were found to be accepted. This indicates that there is a connection between organizational performance and creative behaviors, innovative behaviors, proactive behaviors, autonomy, and risk sharing. On the other hand, news media acts as a mediator between creativeness, innovativeness, proactiveness, autonomy, risk sharing, and organizational performance. These findings are consistent with the findings of Cui et al. (2018), who stated that entrepreneurial orientation has an effect on news media, and news media changes the thoughts of people, which in turn has an effect on the performance of organizations. These findings correspond to the findings of Zahra (2008). It is presumed that the level of risk taking is more on the moderate side rather than the high side because it is possible that higher levels of risk taking could lead to the collapse of such set ups as well. In a similar vein, creativeness, proactiveness, and autonomy are all factors that boost the performance of such set-ups. This is because these characteristics have become essential for entrepreneurs to possess in order to thrive in an unstable environment.

It was hypothesized in H12 that public opinion played a moderating role in the connection between the news media and the performance of organizations. According to the results of the study, an organization's overall performance can be improved if the people who are affiliated with it, including its employees and members of the public, have a favorable impression of the business (Gorodnichenko et al., 2021). As a result, H12 is also acceptable.

## 6. Implications

According to the findings of the research, an individual's mentality regarding venturing out on their own and beginning their own business has a considerable bearing on the level of success or failure that a small firm has. As was mentioned before, having a solitary focus does not guarantee that one would be successful. Instead, the distribution of tasks and the adoption of shared risks is far more significant, as was proved in the challenging circumstances of COVID-19. An entrepreneur who has the appropriate perspective has the potential to be the engine that propels the success of their firm. Therefore, it is essential for stakeholders to recognize that entrepreneurship does not entail taking chances or going for a hit-and-try strategy; rather, it is about owning the adequate set of knowledge, skills, and abilities in order to be successful. This understanding is important because entrepreneurialism is not about taking chances or going for a hit-and-try approach. An entrepreneurial activity that is fruitful, particularly in the area of small enterprises, can have beneficial repercussions for a number of different people in addition to businesses as a whole. The study's authors advise policy makers and educational institutions to create mechanisms for training people who have an orientation toward entrepreneurship rather than just encouraging new entrepreneurs by giving grants and funds. This is due to the fact that we are aware of the beneficial effects that an entrepreneurial endeavor can have on a number of people as well as enterprises as a whole. However, for entrepreneurs as a whole, having a vision and confidence might be an advantageous pre-requisite for creating and managing a business. However, a good entrepreneur also needs to have high capabilities, and if an entrepreneur lacks particular competencies, there is no wrong in recruiting qualified or talented people. Last but not least, learning and training have no boundaries in this context; one can always work to acquire new and improved knowledge in order to achieve greater results, whether for managing oneself or one's own business. Despite this, no one hidden technique, set of abilities, or collection of skills can ensure the success of a small firm. But some of the points made in this study and the related literature can undoubtedly aid existing or aspiring businesspeople with an entrepreneurial orientation in managing or overcoming some tactical or strategic challenges.

## 7. Limitation and future direction

This study, just like any other kind of research, had a few shortcomings in a few key areas.

To begin, the fact that this study was conducted in small and medium-sized enterprises (SMEs) in China raises questions about the degree to which its findings are applicable to a more general population. In the future, researchers may conduct surveys in a number of different countries or regions in order to make findings that are more general. Second, the participants in this study were limited to managers of small and medium-sized enterprises (SMEs), and the data was collected using a convenience sample. Both of these factors limited the generalizability of the findings of our research because they were not representative of the entire population. As a consequence of this, subsequent research might make an effort to collect data from employees of SMEs.

Third, all of the findings in this study are subject to the possibility of biasness because the participants were asked to report their own performance. Even though self-reporting has been used in a large number of studies for a long time and has the benefits of being convenient and inexpensive, future research should make an effort to use a variety of approaches (such as psychological experiments and internet ethnography) and data sources in order to increase the validity of the results. This will allow for a greater understanding of the relationships between the variables being studied. Further research should be conducted to investigate the long-term effects of entrepreneurship on organizational performance in multiple firms.

## 8. Conclusion

Organizational performance is the process through which an organization achieves its goals. This includes all the activities that help an organization meet its objectives and achieve its mission. It also involves the people who work in the organization, especially those who are directly responsible for achieving organizational objectives. The study of how entrepreneurial orientation, risk sharing, and organizational performance are connected is ongoing. In this research, we look at not only the connection between these three factors but also the function that the news media plays as a mediator and the role that public opinion plays as a moderator in this connection. In addition, the findings of this research underline the importance of contextualizing and reevaluating the information and abilities that are being distributed by educational establishments of a higher level. In conclusion, but certainly not least, the findings of this research have made it possible for small firms to better prepare themselves to deal with unforeseen obstacles in order to guarantee their ongoing survival over the long run.

## Data availability statement

The original contributions presented in the study are included in the article/supplementary material, further inquiries can be directed to the corresponding author.

## Ethics statement

The studies involving human participants were reviewed and approved by Shanxi University, China. The patients/participants provided their written informed consent to participate in this study. The study was conducted in accordance with the Declaration of Helsinki.

## Author contributions

ZZ: conceptualization, data collection, and writing the draft. YX: supervision and editing. All author agreed to the submitted version of manuscript.
